# The influence of low-temperature resistant lactic acid bacteria on the enhancement of quality and the microbial community in winter Jerusalem Artichoke (*Helianthus tuberosus L.*) silage on the Qinghai-Tibet Plateau

**DOI:** 10.3389/fmicb.2024.1297220

**Published:** 2024-01-29

**Authors:** Xiaoqiang Wei, Xuemei Sun, Haiwang Zhang, Qiwen Zhong, Guangxin Lu

**Affiliations:** ^1^Qinghai University, Xining, China; ^2^Qinghai Provincial Key Laboratory of Vegetable Genetics and Physiology, Xining, China; ^3^Academy of Agriculture and Forestry Sciences, Qinghai University, Xining, China; ^4^College of Agriculture and Animal Husbandry, Qinghai University, Xining, China

**Keywords:** Qinghai-Tibet Plateau, Jerusalem Artichoke silage, low-temperature resistant lactic acid bacteria, winter fermentation, microbial community structure

## Abstract

Jerusalem Artichoke (*Helianthus tuberosus L.*), an emerging “food and fodder” economic crop on the Qinghai-Tibet Plateau. To tackle problems such as incomplete fermentation and nutrient loss occurring during the low-temperature ensilage of Jerusalem Artichokes in the plateau’s winter, this study inoculated two strains of low-temperature resistant lactic acid bacteria, *Lactobacillus plantarum* (GN02) and *Lactobacillus brevis* (XN25), along with their mixed components, into Jerusalem Artichoke silage material. We investigated how low-temperature resistant lactic acid bacteria enhance the quality of low-temperature silage fermentation for Jerusalem Artichokes and clarify its mutual feedback effect with microorganisms. Results indicated that inoculating low-temperature resistant lactic acid bacteria significantly reduces the potential of hydrogen and water-soluble carbohydrates content of silage, while increasing lactic acid and acetic acid levels, reducing propionic acid, and preserving additional dry matter. Inoculating the *L. plantarum* group during fermentation lowers pH and propionic acid levels, increases lactic acid content, and maintains a dry matter content similar to the original material. Bacterial community diversity exhibited more pronounced changes than fungal diversity, with inoculation having a minor effect on fungal community diversity. Within the bacteria, *Lactobacillus* remains consistently abundant (>85%) in the inoculated *L. plantarum* group. At the fungal phylum and genus levels, no significant changes were observed following fermentation, and dominant fungal genera in all groups did not differ significantly from those in the raw material. *L. plantarum* exhibited a positive correlation with lactic acid and negative correlations with pH and propionic acid. In summary, the inoculation of *L. plantarum* GN02 facilitated the fermentation process, preserved an acidic silage environment, and ensured high fermentation quality; it is a suitable inoculant for low-temperature silage in the Qinghai-Tibet Plateau.

## 1 Introduction

Qinghai Province is situated in the northeastern region of the Qinghai-Tibet Plateau. The growth of animal husbandry has been significantly impeded by insufficient grass during the cold season and other factors. Planting ‘food and fodder’ cash crops to augment the forage supply is considered an effective approach to address this issue (Xin, 2013; Qi, 2021). Jerusalem Artichoke (JA, *Helianthus tuberosus L.*), a ‘food and fodder’ cash crop cultivated for its tubers. It exhibits characteristics such as cold tolerance (Zhao et al., 2020), drought resistance (Zhou et al., 2022), salinity resistance (Li et al., 2016; Teng, 2019), disease resistance, and robust stress resilience (Qiu et al., 2018). It thrives in adverse growth conditions, including wasteland, saline-alkali soil, and gravel. The crop boasts a high yield of aboveground stems and leaves, enriched nutritional value, and favorable palatability. The feed value of its stems and leaves surpasses that of *Solanum tuberosum* and *Helianthus annuus*, rendering it a superior-quality roughage featured in the China Feed Database^1^ (Wang et al., 2020a,b). Research has demonstrated that JA tubers, stems, and leaves are abundant in fructans. When used as dietary additives, they can significantly enhance the intestinal microorganisms of ruminants, bolster lactation performance in dairy cows, and lower the occurrence of mastitis in dairy cows (Wang et al., 2021a,b,c).

Silage involves the storage of feed through lactic acid bacteria (LAB) fermentation (Ren et al., 2021; Okoye et al., 2023). The post-fermentation lactic acid, dry matter, and ammonia nitrogen contents of JA all align with the benchmarks for high-quality silage (Monllor et al., 2020). Ambient temperature is an important factor affecting the quality of silage (Zhou et al., 2016). Ordinarily, JA silage undergoes fermentation at room temperature for 40–45 days (Wu et al., 2022). However, the average daily temperature during JA harvesting in Qinghai Province hovers around 10°C, occasionally plummeting to below 0°C at night. The fermentation process is inhibited at low temperatures (Li et al., 2021, 2022). In colder temperatures (<5°C), inoculating with low-temperature resistant LAB can expedite the low-temperature fermentation process, inhibit the proliferation of detrimental microorganisms, and substantially enhance silage quality. Li (2016) employed corn (*Zea mays*) and alfalfa (*Lotuscorniculatus*) as silage substrates and inoculated three low-temperature resistant LAB at 15°C. This intervention resulted in reduced pH and ammonia nitrogen content and a noteworthy elevation in silage lactic acid content, and enhanced fermentation. Consequently, the inoculation of low-temperature resistant LAB can enhance the low-temperature ensiling of Jerusalem Artichokes on the Qinghai-Tibet Plateau. Research indicates that particular LAB strains, isolated from silage within distinct environmental contexts, could be optimal for enhancing silage fermentation quality (Ju, 2020; Lin et al., 2022). Initially in this study, we isolated high acid-producing, fast-growing, and low-temperature resistant strains—*Lactobacillus plantarum* GN02 and *Lactobacillus brevis* XN25—from JA silage on the Qinghai-Tibet Plateau, utilized for both homotypic and heterotypic LAB fermentation (Wei et al., 2023). Homotypic fermentation LAB rapidly generates lactic acid throughout fermentation, thereby lowering the silage pH and creating an acidic milieu. In contrast, heterotypic fermentation LAB generates both lactic acid and acetic acid during silage fermentation, enhancing silage’s aerobic stability (Xu et al., 2021; Liu et al., 2022). Nonetheless, the current understanding of how the mechanisms and microbial progression of homotypic and heterotypic fermentation by low-temperature resistant LAB influence the low-temperature silage fermentation of JA remains incomplete. Furthermore, effective assessment criteria for JA silage are lacking. The quality of low-temperature silage fermentation is influenced by microbial community structure and abundance. The elevated presence of harmful bacterial species and the inadequate representation of beneficial bacteria during silage, thereby contributing to fermentation failure (Cheng et al., 2021). Previous studies have highlighted the contribution of bacterial community to silage fermentation. However, Li et al. (2021) reported that *Saccharomyces* and spoilage fungi can impact silage fermentation quality by depleting water-soluble carbohydrates and lactic acid, alongside mycotoxin generation and pH elevation. It is unclear how the response of microbial community of JA silage to the inoculation of low-temperature resistant LAB.

This study utilized JA stems and leaves as experimental materials. They were inoculated with homotypic fermentation and heterotypic fermentation low-temperature resistant LAB during silage preparation in a natural low-temperature environment. The impact of low-temperature resistant LAB on the fermentation quality of JA low-temperature silage was assessed by tracking fermentation parameter changes. Additionally, high-throughput sequencing was employed to unveil the microbial succession throughout the fermentation process, elucidating the dominant species within JA silage and their correlation with fermentation products. This exploration aims to elucidate the regulatory mechanism of low-temperature resistant LAB on JA silage. The findings of this study offer a theoretical foundation for developing JA silage on the Qinghai-Tibet Plateau, providing substantial guidance for the sustainable and stable advancement of animal husbandry in this region.

## 2 Materials and methods

### 2.1 Silage preparation

Test materials were sourced from the Academy of Agriculture and Forestry Sciences at Qinghai University. The JA variety ‘QingYu No. 2’ was selected and cultivated in the experimental field of the Academy of Agriculture and Forestry Sciences of Qinghai University, Xining, Qinghai Province, China. Planting took place on early April 20, 2022, at geographical coordinates E101 °44 ′57.7572 ″, N36 °43 ′8.346 ″, and an elevation of 2266.6 m. On October 21, 2022, the above-ground stems and leaves were crushed to a length of 2 ± 1 cm and utilized as raw materials for JA silage production. The low-temperature resistant LAB employed in the experiment was derived from *L. plantarum* GN02 and *L. brevis* XN25, which were initially selected in the laboratory (Wei et al., 2023). The NCBI registration numbers for these strains were OP740787 and OP740791, respectively. Viable bacterial counts were determined using OD values and centrifugation (4,000 rpm, 5 min). The resulting bacterial pellet was resuspended in sterile water to achieve the number of viable bacteria 1 × 10^8^ cfu/mL, creating a bacterial solution for backup. A 500 g sample of crushed JA raw materials was selected at random. The prepared LAB inoculant was manually and uniformly sprayed onto the raw materials using a sterile watering can (Inoculation dose is 1.0 × 10^6^ cfu/g of FM.). After thorough mixing, the mixture was placed in a vacuum-sealed polyethylene plastic bag (280 mm × 350 mm, Zhejiang, China). The bag was vacuum-sealed using a vacuum machine (model 14,891, Deli, Shandong, China), and the mixture was then stored in a natural environment for fermentation. The experiment comprised 4 distinct treatments: CK (sterile water, 5 mL), FD (*L. brevis* XN25, 1.0 × 10^6^ cfu/g of FM), FZ (*L. plantarum* GN02, 1.0 × 10^6^ cfu/g of FM), and FZD (*L. brevis* XN25 and *L. plantarum* GN02 were mixed, 1.0 × 10^6^ cfu/g of FM). Each treatment was replicated three times and bags were opened for analyses after 7 and 60 days of ensiling. In total, 27 bags were sampled (2 storage periods × 4 treatments × 3 replicates +3 raw materials). Silage samples were collected to assess fermentation parameters and microbial communities.

### 2.2 Measurement of fermentation parameters

Raw materials and silage samples are analyzed for pH, dry matter, crude protein, water-soluble carbohydrates, neutral detergent fiber, and acid detergent fiber content. Silage samples additionally necessitate assessing lactic acid, acetic acid, and propionic acid levels. Homogenize 20 g of the sample with 180 mL of distilled water in a high-speed juicer for 30 s. Filter the mixture through 4 layers of medical gauze to collect the filtrate. Centrifuge the filtrate at 5,000 rpm for 10 min. Measure the pH of the filtrate using a glass electrode pH meter (pH S-2F, LEICI, Shanghai, China). Store the filtrate at −20°C for subsequent analysis of lactic acid (LA), acetic acid (AA), propionic acid (PA), and ammonia nitrogen (NH_3_-N) content. LA, AA, and PA contents were quantified using high-performance liquid chromatography (Li et al., 2018). NH_3_-N content was extracted using the Plant Ammonia Nitrogen Content Assay Kit (Beijing Boxbio Science & Technology Co., Ltd.) and measured at 570 nm using a microplate reader (EPOCH2, BioTek, Vermont, USA). Another 300 g sample was dried at 65°C in an oven for 48 h until a constant weight was achieved. The dry matter (DM) content was determined using an electronic balance (PTX-FA210S, HuaZhi, Connecticut, USA). The dried sample was ground using a grinder and sieved through a 1 mm screen. The resulting sample was utilized for quantifying crude protein (CP), water-soluble carbohydrates (WSC), neutral detergent fiber (NDF), and acid detergent fiber (ADF) content. CP content was quantified using a Kjeldahl nitrogen analyzer (K9860, Hainon, Shandong, China) following the analytical method outlined by the Society of Chemists (AOAC, 1990). WSC content analysis was conducted using the anthrone reagent colorimetric method (Thomas, 1977). NDF and ADF contents were measured using a fiber analyzer (F800, Hainon, Shandong, China) following the methods described by Van Soest et al. (1991).

### 2.3 Microbial community analysis

Immediately after collection, silage samples were rapidly frozen and stored at −80°C. Bacterial and fungal DNA were extracted from the silage using a MagPure Soil DNA LQ Kit (Yesen, Shanghai, China), in accordance with the manufacturer’s instructions. DNA concentration was quantified using a NanoDrop 2000 spectrophotometer (Thermo Fisher Scientific, Waltham, MA, USA), while integrity was assessed through agarose gel electrophoresis. PCR amplification targeting the V3-V4 hypervariable region of bacterial 16S rRNA and the ITS1 region of fungal ITS gene was conducted in 25 mL reaction volumes, employing universal primer pairs. For bacteria, the primers were 343F (5’-TACGGRAGGCAGCAG-3′) and 798R (5’-AGGGTATCTAATCCT-3′). For fungi, the primers were ITS1F (5’-CTTGGTCATTTAGAGGAAGTAA-3′) and ITS2 (5’-GCTGCGTTCTTCATCGATGC-3′). The reverse primer included a sample-specific barcode, and both primers were linked to an Illumina sequencing adapter.

Amplicon quality was assessed through gel electrophoresis. PCR products were purified using Agencourt AMPure XP beads (Beckman Coulter Co., USA) and quantified with the Qubit dsDNA assay kit. Subsequently, the concentrations were adjusted for sequencing purposes. Sequencing was conducted on an Illumina NovaSeq6000 using two paired-end read cycles of 250 bases each (Illumina Inc., San Diego, CA; OE Biotech Company, Shanghai, China). The raw sequencing data were in FASTQ format. Subsequently, paired-end reads underwent preprocessing using cutadapt software to identify and remove adapters. Following trimming, the paired-end reads were filtered for low-quality sequences, denoised, merged, and subjected to chimera detection and removal using DADA2 (Callahan et al., 2016) with QIIME2’s default parameters (Bolyen et al., 2019). Finally, the software generated representative reads and an abundance table for ASVs. The representative read for each ASV was chosen utilizing the QIIME 2 package. These representative reads were then annotated and compared to the Silva database Version 138 (16 s rDNA) and Unite (ITS) using q2-feature-classifier with the default parameters. Microbial diversity within the silage samples was assessed using alpha diversity metrics, including the Chao1 index (Chao and Bunge, 2002) and Shannon index (Hill et al., 2003). The Unifrac distance matrix, computed using QIIME software, was employed for unweighted UniFrac principal coordinates analysis (PCoA). Bacterial differential markers were identified using LEfSe (LDA) analysis, employing an LDA Score screening threshold of 4 (Weiss, 2017).

### 2.4 Statistical analysis

We utilized Excel 2019 for fundamental statistical analysis. Additionally, we employed IBM SPSS 27.0 for two-way analysis of variance, including Duncan’s multiple comparisons, to assess fermentation attributes and chemical parameters. A value of *p* < 0.05 was considered statistically significant, and a value of *p* < 0.01 was considered very significant. We analyzed and plotted microbial sequencing data on the Oebiotech Cloud Platform.^2^

## 3 Results

### 3.1 Fermentation attributes of the silage samples

As shown in Table 1, throughout the ensiling fermentation period, pH levels in all groups decreased relative to F0. Significant pH differences were observed among groups during ensiling. After 60 days of ensiling, only FZ silage had pH values lower than 5.0, whereas the pH values of other silages remained at high levels (values for CK and FD silages). LA content exhibited an upward trajectory over time in the CK and FD groups, in contrast to the decreasing trend observed in the FZ and FZD groups, aligning with the pH pattern. Substantial variations in LA content were evident among groups on 7, 60 days. Notably, the FZ group exhibited the highest LA content on both 7, 60 days (26.93 g/kg and 24.09 g/kg FM). In contrast, the CK group had the lowest LA content (0.57 g/kg and 4.08 g/kg FM) on the respective days. Gradual AA content increments were observed across all groups over time. Specifically, on 7, 60 days, the FD group demonstrated notably elevated AA content in comparison to the remaining groups (11.66 g/kg and 16.37 g/kg FM). Conversely, PA content exhibited a gradual decline across all groups over time. On 7, 60 days, the PA content of the CK group exhibited significant elevation in comparison to the other groups, whereas the FZ group demonstrated considerable reduction (2.15 g/kg and 2.17 g/kg FM). The FZD group exhibited significantly higher NH_3_-N content compared to the others. Interestingly, on 60 days, the FD group demonstrated a marked rise in NH_3_-N content (17.61 g/kg FM), significantly surpassing the other groups. Two-way analysis of variance indicates noteworthy interactions (*p* < 0.01) among the fermentation attributes (pH, LA, AA, PA, NH_3_-N) concerning fermentation period and microbial strains.

**Table 1:** The quality of silage fermentation undergoes dynamic changes.

Item	Fresh	Significance	Treatment	Days		SEM	*p* value		
	F0			**7**	60		T	D	T × D
pH	8.18	A	CK	6.20bC	6.55aB	0.02	0.001	0.001	0.001
		A	FD	6.48aB	5.18cC				
		A	FZ	4.81 dB	4.53dC				
		A	FZD	5.62cB	5.33bC				
LA (g/kg FM)	-	-	CK	0.57d	4.08d	0.221	0.001	0.796	0.001
		-	FD	2.62c	6.46c				
		-	FZ	26.94a	24.09a				
		-	FZD	20.42b	15.76b				
AA (g/kg FM)	-	-	CK	0.45c	2.26c	0.442	0.001	0.001	0.001
		-	FD	11.66a	16.37a				
		-	FZ	1.66c	1.67c				
		-	FZD	7.07b	10.82b				
PA (g/kg FM)	-	-	CK	6.05a	3.54a	0.161	0.001	0.001	0.001
		-	FD	4.17b	2.21b				
		-	FZ	2.15c	2.17b				
		-	FZD	3.71b	3.08a				
NH_3_-N (μg/mL FM)	9.71	A	CK	7.28bB	8.47cAB	0.459	0.001	0.001	0.001
		B	FD	8.55bB	17.61aA				
		A	FZ	7.57bB	9.65cA				
		B	FZD	10.66aB	13.05bA				

pH, potential of hydrogen; LA, lactic acid; AA, acetic acid; PA, propionic acid; NH_3_-N, ammonia nitrogen; F0, JA raw material; CK, sterile water; FD, *Lactobacillus brevis*; FZ, *Lactobacillus plantarum*; FZD, the combination of *L. brevis* and *L. plantarum*. Means with different letters in the same row or column indicate a significant difference according to Duncan test (*p* < 0.05). SEM, standard error of means; T, treatment; D, ensiling days; T × D, interaction between treatment and ensiling days.

### 3.2 Chemical parameters for both raw materials and silage samples

As shown in Table 2, throughout the ensiling fermentation period, the DM contents of each group exhibited a progressive decrease. On 7 days, no noteworthy disparities in DM contents were observed among the groups. The CK group displayed a swifter decline during fermentation and exhibited a notably lower level than the other groups by 60 days. The DM content of the FZ group at the 60 days mark (333.17 g/kg) was markedly greater than that of the CK and FZD groups. The WSC content of all groups exhibited a notable decrease over time, and it was significantly lower than that of the initial state (F0). Throughout fermentation, the FD group displayed elevated WSC content (24.83 g/kg, 12.85 g/kg DM) in comparison to the other groups, yet no significant difference was observed between FD and FZ on 60 days. The CP content of all groups experienced a slight increase over time, and the FZD group exhibited significant elevation over the others on 7 days; no significant differences were detected among the groups on 60 days. Compared to the F0 group, the NH_3_-N content decreased in all groups except for the FZD group on 7 days. The NDF content of all groups saw an increase relative to the F0 state, and the FZ group exhibited both the highest content and significant elevation on 7 days. No significant differences were observed among the groups on 60 days. Analogous to the NDF pattern, the ADF content of all groups showed an increase in relation to the F0 state, with the FZ and FZD groups displaying higher content on 60 days. Intriguingly, on 7, 60 days, no significant differences were found between the CK group and the FZ or FZD groups; all three showed higher values than the FD group. Two-way analysis of variance shows notable disparities (*p* < 0.01) in DM and WSC chemical parameters among fermentation periods and microbial strains. However, the interaction between them is not statistically significant (*p* > 0.05), suggesting a lack of interaction between fermentation periods and microbial strains in DM and WSC. On the contrary, a significant interaction (*p* < 0.01) is observed among the chemical parameters of CP, NDF, and ADF across fermentation periods and microbial strains.

**Table 2:** The chemical composition of silage fermentation undergoes dynamic fluctuations.

Item	Fresh	Significance	Treatment	Days		SEM	*p* value		
	F0			7	60		T	D	T × D
DM (%)	35.02	A	CK	33.21aB	31.00cC	0.35	0.003	0.001	0.077
		A	FD	33.47aB	32.69aB				
		A	FZ	34.18aAB	33.32aB				
		A	FZD	34.11aA	31.84bB				
WSC (g/kg DM)	61.21	A	CK	20.95abB	9.33cC	0.917	0.001	0.001	0.133
		A	FD	24.83aB	12.85aC				
		A	FZ	19.54bB	10.97bC				
		A	FZD	17.53bB	9.08cC				
CP (% DM)	10.31	C	CK	11.52abA	10.97aB	0.256	0.001	0.015	0.015
		A	FD	10.32cA	10.83aA				
		A	FZ	10.81bcA	10.27bA				
		C	FZD	12.38aA	10.97aB				
NDF (% DM)	38.13	C	CK	40.40cB	41.87aA	0.866	0.002	0.83	0.001
		B	FD	39.40cA	40.07aA				
		B	FZ	46.53aA	41.00aB				
		B	FZD	39.33cB	42.20aA				
ADF (% DM)	28.00	A	CK	36.20aB	37.20aB	1.057	0.001	0.913	0.001
		B	FD	34.07aA	28.60bB				
		B	FZ	35.33aA	38.87aA				
		C	FZD	33.93aB	38.93aA				

DM, dry matter; WSC, water-soluble carbohydrates; CP, crude protein; NDF, neutral detergent fiber; ADF, acid detergent fiber; F0, JA raw material; CK, sterile water; FD, *L. brevis*; FZ, *L. plantarum*; FZD, the combination of *L. brevis* and *L. plantarum*. Means with different letters in the same row or column indicate a significant difference according to Duncan test (*p* < 0.05). SEM, standard error of means; T, treatment; D, ensiling days; T × D, interaction between treatment and ensiling days.

### 3.3 Analysis of microbial community diversity in both raw materials and silage samples

**Figure 1:**
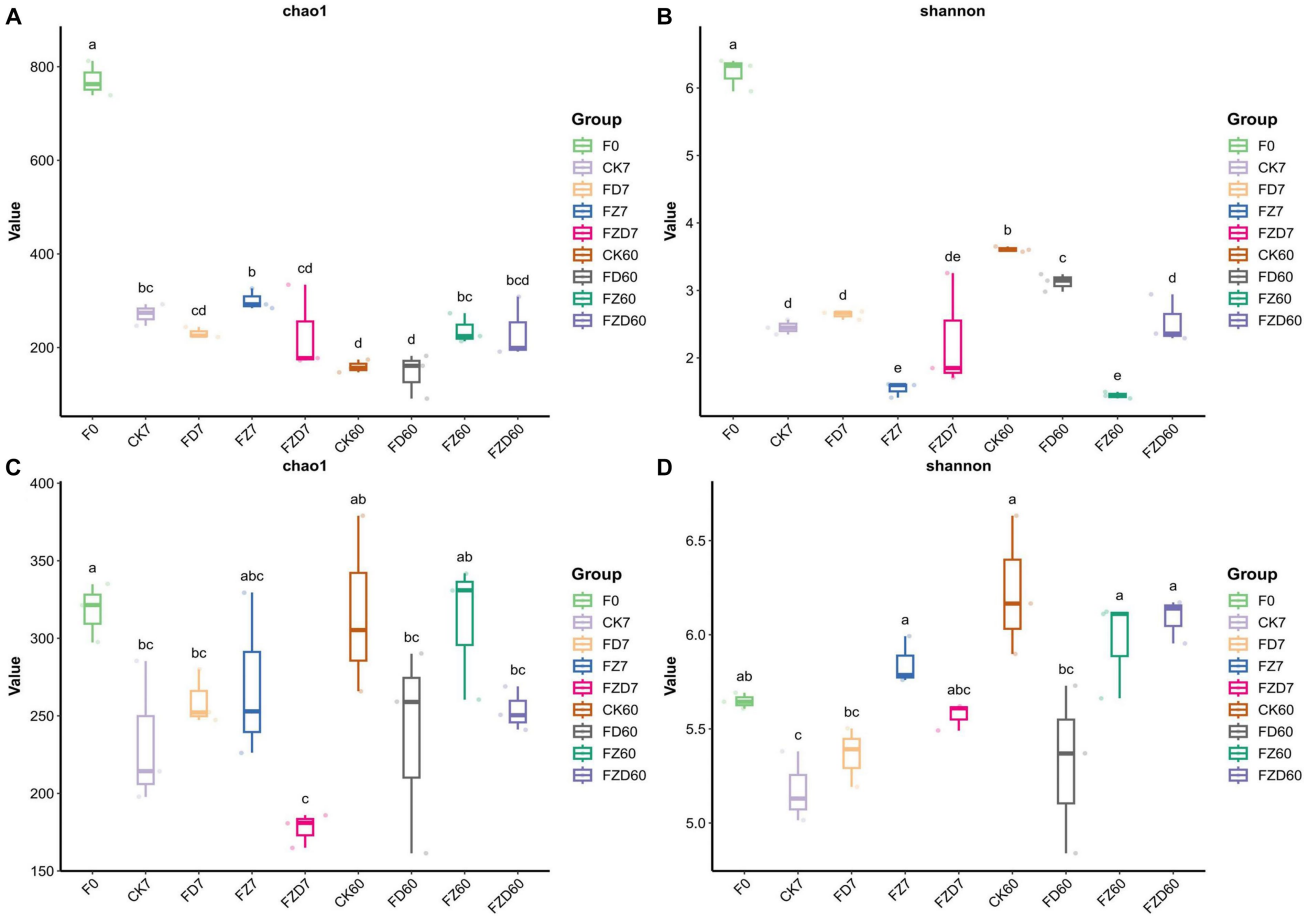
The Chao1 and Shannon indices represent the α-diversity of bacteria **(A,B)** and fungi **(C,D)**. F0, JA raw material; CK, sterile water; FD, *Lactobacillus brevis*; FZ, *Lactobacillus plantarum*; FZD, the combination of *L. brevis* and *L. plantarum*; The numbers 7 and 60 correspond to fermentation duration; The use of different lowercase letters signifies statistically significant differences (*p* < 0.05) among various treatments on either the same or different ensiling days, while treatments sharing the same lowercase letters indicate no statistically significant differences (*p* > 0.05) between them.

We use the Chao1 and Shannon indices of α-diversity to describe the abundance and diversity of bacterial and fungal populations within groups. The Chao1 index estimates the number of ASVs in the community, while the Shannon index estimates intragroup community diversity. Higher values for both indices suggest greater richness and diversity within intragroup communities. In bacteria (Figures 1A,B), the Chao1 (771.73) and Shannon (6.23) indices of the F0 group were significantly higher than those of the other groups. On 7 days, the Chao1 index of the FZ group (301.24) was significantly higher than that of the FZD group (227.77), while the Chao1 indices of the other groups showed no significant differences. Moreover, the Shannon indices of the CK and FD groups (2.45, 2.64) were significantly higher than that of the FZ group (1.54), and there were no significant differences from the FZD group. These findings indicate that the FZD group exhibited the lowest community richness on 7 days, whereas the FZ group had the lowest community diversity. On 60 days, the Chao1 indices of the FZ (237.31) and FZD (233.11) groups were significantly higher than those of the CK (159.61) and FD (144.87) groups and no significant differences were observed within the CK, FD, and FZ, FZD groups. Additionally, the Shannon index of the FZ group (1.44) was significantly lower than that of the other groups, with no significant differences observed among the other groups. These findings suggest that the CK and FD groups exhibited the lowest community richness on 60 days, while the FZ group had the lowest community diversity. With the progression of fermentation, variations in the Chao1 index were evident within the identical groups on 7, 60 days. Specifically, the Chao1 indices of the CK groups exhibited a substantial decrease over time, whereas those of the FZD groups displayed a significant increase. No noteworthy effects were observed in the other groups. Similarly, the Shannon index indicated no significant distinctions among the identical groups on 7, 60 days. Within fungi (Figures 1C,D), the Chao1 indices of all groups exhibited no noteworthy variances on fermentation 7, 60 days, suggesting minimal discrepancies in fungal community richness during the fermentation phase. Additionally, the Shannon index indicated no significant distinctions among the groups on 7 days, with no notable differences observed between the groups and the F0 group. However, on 60 days, the CK (6.26) and FZD (6.09) groups demonstrated a substantial elevation compared to the FD (5.31) group. Although the FD group did not display significant differences when compared to the groups on 0 and 7 days, this suggests alterations in fungal community diversity during the later stages of fermentation, particularly evident in the CK and FZD groups.

**Figure 2:**
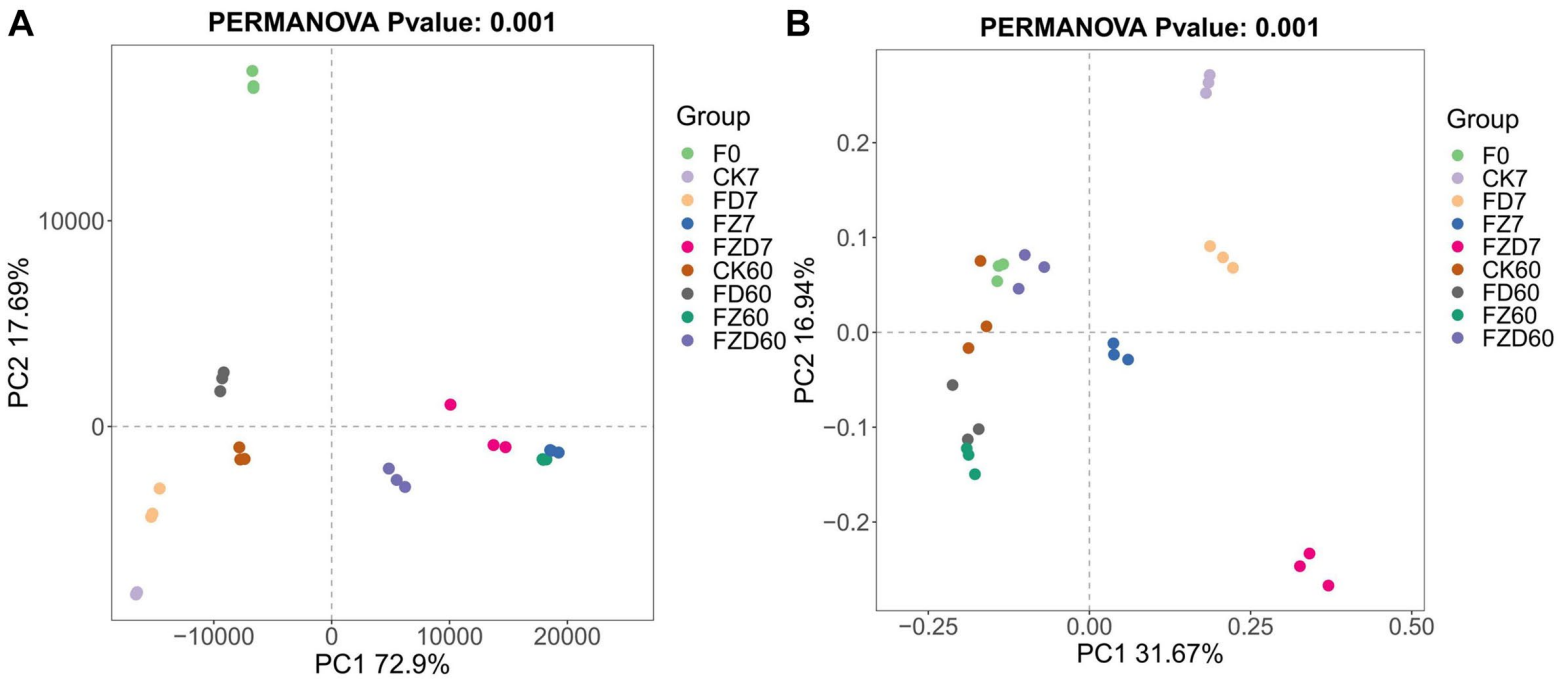
PCoA examines the community structure of bacteria [**(A)** Euclidean method] and fungi [**(B)** Bray curtis method]. F0, JA raw material; CK, sterile water; FD, *L. brevis*; FZ, *L. plantarum*; FZD, the combination of *L. brevis* and *L. plantarum*; The numbers 7 and 60 correspond to fermentation duration; ANOSIM test (*p* < 0.05).

PCoA was performed to analyze the microbial community changes in JA silage during fermentation. Within bacteria (Figure 2A), the F0 group showed substantial separation along the PC2 axis from other groups, while the remaining groups demonstrated dispersed distribution, signifying noteworthy differences between the groups. On 7, 60 days, the CK and FD groups cluster together along the PC1 axis, similar to the clustering of the FZ and FZD groups, emphasizing how the presence of added microbial species significantly contributes to distinct inter-group bacterial variations. Importantly, data for the FZ group at 7, 60 days exhibited substantial overlap. Among fungi (Figure 2B), all groups were segregated along the PC2 axis, and a more pronounced separation was noticeable among the 7 days groups than the 60 days groups. ANOSIM tests utilizing Euclidean distance revealed that differences in bacterial and fungal community structures between groups during fermentation were markedly more substantial than differences within groups (R2 = 0.98, *p* = 0.001; R2 = 0.99, *p* = 0.001), affirming the robustness of PCoA clustering. Therefore, it is clear that the introduction of microbial species impacts alterations in the microbial community throughout the fermentation of JA silage.

### 3.4 Composition of microbial species in both raw materials and silage samples

We conducted high-throughput sequencing on 27 samples utilizing the Illumina MiSeq sequencing platform. Following sequencing, the distribution of raw bacterial reads ranged from 78,045 to 81,711, and after quality control, the distribution of clean tags ranged from 58,290 to 75,142. After eliminating chimeras from the clean tags, valid tags (the data used for analysis) spanned from 44,672 to 70,842. ASV counts per sample varied between 91 and 813. Using 100% sequence similarity, all sequences were classified, yielding a total of 25 phyla, 48 classes, 128 orders, 215 families, 477 genera, and 883 species. Regarding fungi, the distribution of raw reads data spanned from 78,201 to 81,996, clean tags data distribution ranged from 67,322 to 78,183, and valid tags data distribution ranged from 67,322 to 78,064. After classification, ASVs ranged from 152 to 378, resulting in a total of 13 phyla, 33 classes, 72 orders, 162 families, 362 genera, and 508 species.

**Figure 3:**
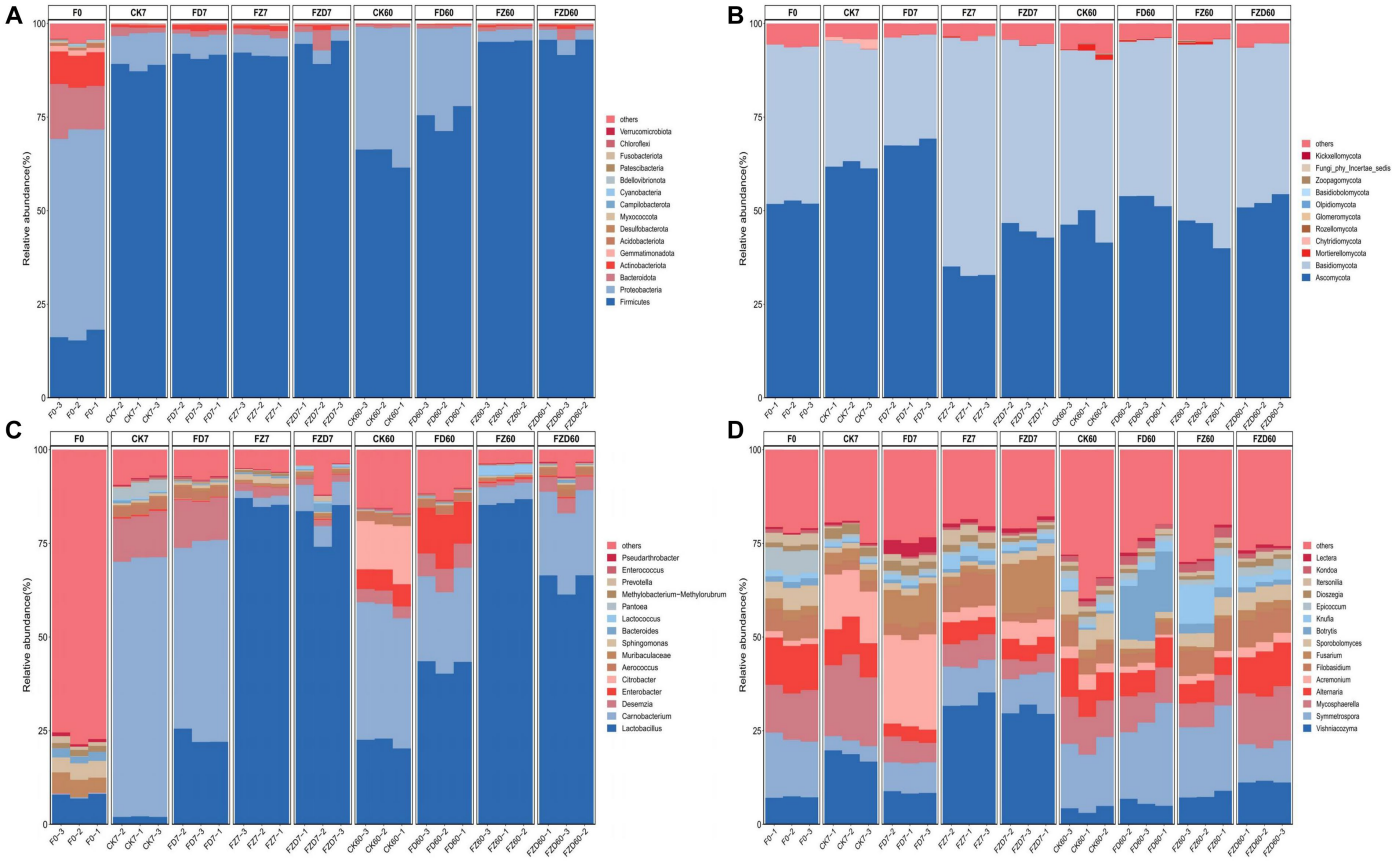
During silage fermentation, the relative abundance of bacteria **(A,C)** and fungi **(B,D)** is assessed at both the phylum and genus levels. F0, JA raw material; CK, sterile water; FD, *L. brevis*; FZ, *L. plantarum*; FZD, the combination of *L. brevis* and *L. plantarum*; The numbers 7 and 60 correspond to fermentation duration.

In bacterial classification at the phylum level (Figure 3A), the species composition of the F0 group distinguishes it from the other groups. The dominant phyla within it are Proteobacteria, Firmicutes, Bacteroidota, and Actinobacteriota, comprising relative abundances of 54.26, 16.59, 12.48, and 8.74%, respectively, contributing to a total community composition of 92.07%. On the 7 days of fermentation, Firmicutes (88.48, 91.37, 91.61, 93.07%) were the predominant phylum in all groups, constituting over 85% of the total. By the 60 days of fermentation, the CK and FD groups were characterized by dominant Firmicutes (64.71, 74.91%) and Proteobacteria (34.24, 23.92%), while the FZ and FZD groups exhibited Firmicutes (95.22, 94.38%) as the prevailing phylum. The evolution of ensiling fermentation reveals shifts in the species composition at the phylum level within each group compared to 0 day. On 7 days, Firmicutes’ relative abundance markedly surged in all groups, establishing it as the prevailing phylum, while the relative abundances of Proteobacteria, Bacteroidota, and Actinobacteriota conspicuously declined, leading to an overall relative abundance falling below 3%. On 60 days, the CK and FD groups exhibited a notable increase in the relative abundance of Proteobacteria, whereas the relative abundance of Firmicutes decreased by 23.76 and 16.46%, respectively. In the FZ and FZD groups, Firmicutes showed a slight increase, maintaining its dominant status at the phylum level.

At the fungal phylum level (Figure 3B), in contrast to bacteria, the dominant phyla were Ascomycota and Basidiomycota across all groups throughout each period, comprising over 90% of the total community. As fermentation progressed, the relative abundances of the two phyla experienced alterations. In comparison to the F0 group, the CK and FD groups showed an initial increase followed by a decrease in the relative abundance of Ascomycota at both 7, 60 days, whereas the relative abundance of Basidiomycota exhibited an initial decrease followed by an increase. Conversely, the FZ and FZD groups demonstrated trends opposite to those of the CK and FD groups in terms of the relative abundance changes of Ascomycota and Basidiomycota. In both time periods, the FD group exhibited the highest relative abundance of Ascomycota (68.03, 53.07%), whereas the FZ group showed the highest relative abundance of Basidiomycota (62.59, 50.22%).

Significant differences in bacterial and fungal genus composition exist among the groups at the genus level. In bacteria (Figure 3C), the relatively abundant genera are *Lactobacillus* (7.66%), *Muribaculaceae* (4.68%), and *Sphingomonas* (4.28%). By the 7 days of fermentation, dominant genera had begun to emerge in all groups. In the CK and FD groups, the dominant genera included *Carnobacterium* (68.82, 51.96%), *Desemzia* (11.63, 11.52%), and *Lactobacillus* (2.05, 23.18%), while *Lactobacillus* was comparatively less abundant in the CK group. Conversely, in the FZ and FZD groups, *Lactobacillus* emerged as the dominant genus (85.70, 81.03%). On 60 days, alterations in dominant genera were evident across all groups. In the CK group, the relative abundance of *Carnobacterium* (35.78%) and *Desemzia* (3.41%) decreased, while *Lactobacillus* (21.90%) increased. This period also saw the emergence of *Citrobacter* (13.52%) and *Enterobacter* (5.61%). In the FD group, the relative abundance of *Lactobacillus* (42.40%) increased, while *Carnobacterium* (23.17%) and *Desemzia* (6.24%) declined. *Enterobacter* (12.65%) began to appear. The FZ group maintained *Lactobacillus* (85.94%) as the dominant genus, experiencing minimal changes in relative abundance. Conversely, the FZD group witnessed a decrease in *Lactobacillus* (64.84%) alongside an increase in *Carnobacterium* (22.26%) and a slight presence of *Desemzia* (3.89%).

At the genus level of fungi (Figure 3D), in the F0 group, the relatively abundant genera are Symmetrospora (15.77%), Mycosphaerella (12.96%), Alternaria (12.51%), and Vishniacozyma (7.28%). On 7 days, the CK group experienced an increase in the relative abundance of *Vishniacozyma* (18.4%), *Mycosphaerella* (20.07%), and *Acremonium* (13.61%), which became dominant genera. Similarly, the FD group saw *Acremonium* (24.04%) emerge as a dominant genus, while in the FZ and FZD groups, the dominant genus is *Vishniacozyma* (32.90, 30.32%). On 60 days, shifts in genus abundance occurred, with *Symmetrospora*, *Mycosphaerella*, *Alternaria*, and *Vishniacozyma* emerging as dominant genera in each group. Notably, these genera showed relatively uniform distribution among groups.

### 3.5 Correlation analysis between measured variables and microbial community

**Figure 4:**
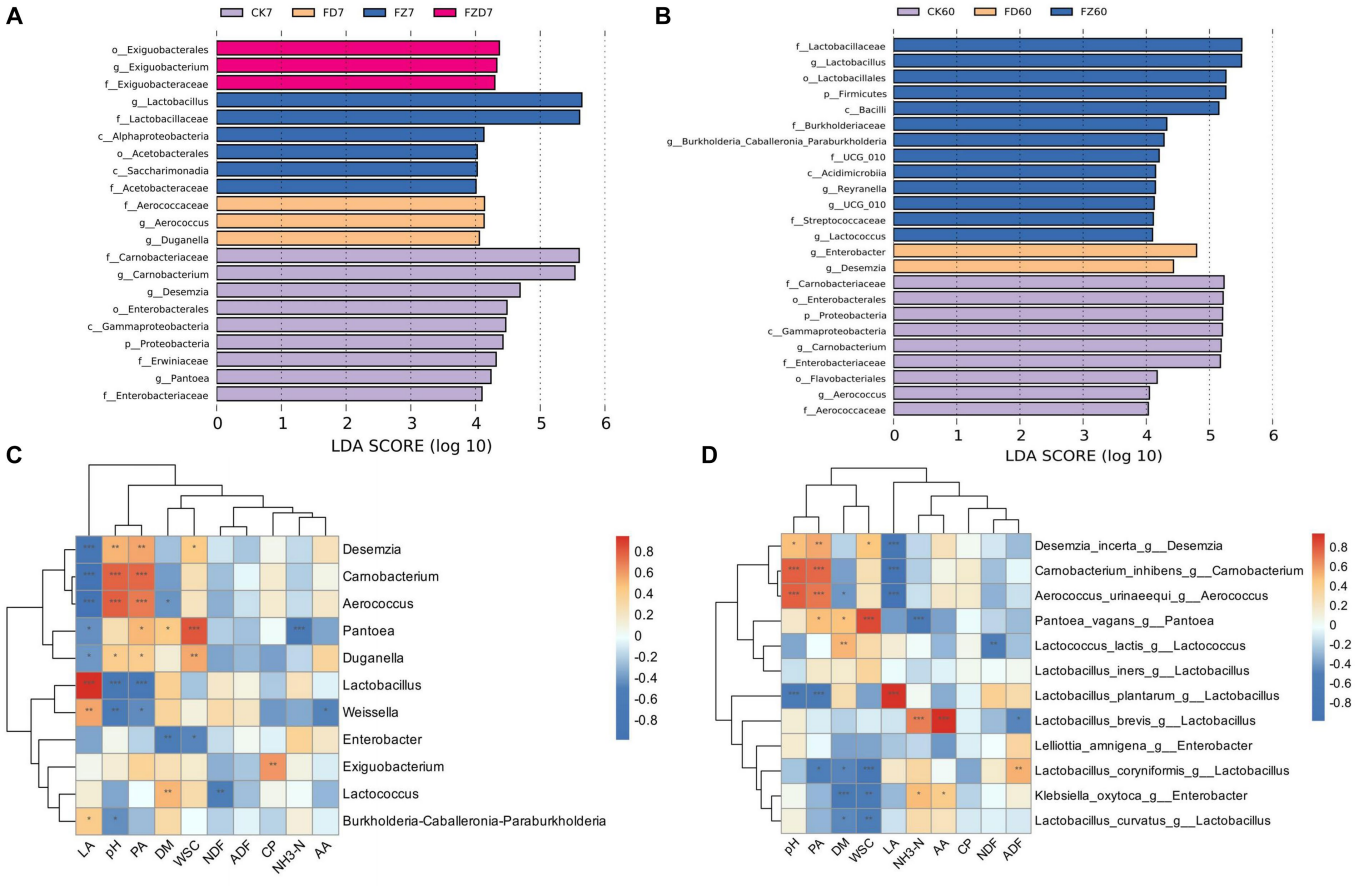
LEfSe analysis results for bacterial genus-level differential biomarkers on both the 7 d **(A)** and the 60 d **(B)** of ensiling fermentation (LDA score = 4). CK, sterile water; FD, *L. brevis*; FZ, *L. plantarum*; FZD, the combination of *L. brevis* and *L. plantarum*; The numbers 7 and 60 correspond to fermentation duration. The Spearman correlation heatmap **(C)** depicts the correlation between bacterial genus-level differential biomarkers and ensiling fermentation parameters; The analysis in Spearman correlation heatmap **(D)** included the selection of the top 30 species-level differential biomarkers from the bacterial genus-level differential biomarkers and their correlation analysis with fermentation parameters; Significance levels are denoted as *** (*p* < 0.001), ** (*p* < 0.01), and * (*p* < 0.05); red signifies a positive correlation, blue represents a negative correlation, while white indicates no correlation; Decimal values indicate the correlation coefficient R.

LEfSe is a frequently employed tool for analyzing taxa that exhibit significant differences across various taxonomic levels. The preset LDA score threshold for filtering is set at 4. Analysis reveals the enrichment of diverse bacterial species among groups during the fermentation process. On 7 days (Figure 4A), the CK group exhibits notable species enrichment, including *Carnobacterium*, *Desemzia*, and *Pantoea*; the FD group shows enrichment of *Duganella* and *Aerococcus*; the FZ group demonstrates *Lactobacillus* enrichment; the FZD group displays enrichment of *Exiguobacterium*. On 60 days (Figure 4B), the CK group shows significant enrichment of *Carnobacterium* and *Aerococcus*; the FD group displays enrichment of *Enterobacter* and *Desemzia*; the FZ group presents enrichment of *Lactobacillus*, *Burkholderia-Caballeronia-Paraburkholderia*, *Lactococcus*, and *Weissella*; the FZD group does not exhibit distinct enriched taxa.

Correlation heatmaps illustrate the relationship between microbial communities and silage fermentation indicators. Spearman correlation analysis was performed on fermentation indicators, leveraging LEfSe analysis on taxa with notable differences at 7, 60 days, resulting in correlation coefficients (*R* = ±0.6) and significance indexes (*p* < 0.001). Figure 4C reveals that LA exhibits a positive correlation with *Lactobacillus* and a negative correlation with *Desemzia*, *Carnobacterium*, and *Aerococcus*; pH and PA display a positive correlation with *Carnobacterium* and *Aerococcus* while having a negative correlation with *Lactobacillus*; WSC indicates positive correlation with *Pantoea*; NH_3_-N exhibits negative correlation with *Pantoea*; DM, NDF, ADF, CP, and AA show limited correlations with distinct taxa.

To better understand the pivotal taxonomic species affecting fermentation, species that exhibited differential abundances within the top 30 species were chosen for Spearman correlation analysis in relation to fermentation indicators (*R* = ±0.6, *p* < 0.001). As depicted in Figure 4D, LA exhibits a positive correlation with *Lactobacillus plantarum* and negative correlations with *Desemzia incerta*, *Carnobacterium inhibens*, and *Aerococcus urinaeequi*; pH and PA show positive correlations with *A. urinaeequi* and *C. inhibens*, but negative correlations with *L. plantarum*; WSC displays a positive correlation with *Pantoea vagans*, but a negative correlation with *L. coryniformis*; NH_3_-N presents a positive correlation with *L. brevis*, while exhibiting a negative correlation with *P. vagans*; interestingly, at the species level, AA exhibits a positive correlation with *L. brevis*, whereas DM displays a negative correlation with *Klebsiella oxytoca*.

## 4 Discussion

### 4.1 Inoculation enhances the quality of ensiling fermentation of JA

DM and WSC in raw materials are critical for ensuring fermentation quality (Wang et al., 2022). Typically, a DM content between 25–35% and a WSC content exceeding 50 g/kg ensure an ample ensiling fermentation substrate (Hu et al., 2009; Wang et al., 2018). JA raw material has a DM content of 35.02%, surpassing that of corn and oats, and a WSC content of 61.21 g/kg, meeting the criteria for high-quality ensiling material (Chen et al., 2020; Li et al., 2022). The storage temperature significantly influences ensiling success (Zhou et al., 2016). The Tibetan Plateau’s low temperatures make it prone to incomplete ensiling fermentation (Li et al., 2022), but inoculating low-temperature resistant LAB offers a potential solution for winter low-temperature ensiling fermentation (McDonald et al., 1991; Zhou et al., 2016). A pH below 4.6 during ensiling inhibits the activity of undesirable microorganisms and protein hydrolyzing enzymes (Ogunade et al., 2018a,b). In line with prior studies (Chen et al., 2020), inoculating low-temperature resistant LAB results in a gradual pH decrease, correlated with reduced DM and WSC content; microbial metabolism transforms WSC and available nutrients in DM into easily preservable organic acids and other substances (Xia et al., 2022).

Organic acid content is a vital indicator to evaluate fermentation success. Inoculating homotypic fermentation *L. plantarum* increased LA concentration and accelerated feed acidification, aligning with Chen et al.'s (2020) results. Interestingly, in the later fermentation stages, the LA content decreases, possibly due to high raw material WSC content. Homotypic fermentation LAB can produce a substantial amount of LA. As fermentation progresses, excessive WSC consumption shifts the fermentation from homotypic to heterotypic. LA is then converted into CO_2_, ethanol, or AA and volatile fatty acids. This clarifies why the WSC content in *L. plantarum*-inoculated silage is lower than in *L. brevis*-inoculated silage (Li et al., 2018; Bai et al., 2022; Tahir et al., 2023). These gases and volatile components contribute to a decline in ensiled DM and an increase in the relative content of NDF and ADF.

The NH_3_-N content indicates the extent of CP and amino acid decomposition in ensiled feed. Higher values suggest excessive consumption of CP and amino acids, resulting in a decline in silage quality (Hassanat et al., 2007). According to Pahlow et al. (2003) and Wilkinson (2005), proteinase hydrolysis of CP generates NH_3_-N, leading to an elevation in NH_3_-N content and a reduction in CP content in ensiled feed (Guan et al., 2018). At the conclusion of fermentation, there is no significant difference in CP content between groups and the raw material. However, inoculation with *L. brevis* leads to higher NH_3_-N content, possibly due to the elevated pH facilitating the normal activity of protein hydrolyzing enzymes. Moreover, Enterobacteria exhibit increased activity at pH 5–6, generating ammonia and biogenic amines through deamination and decarboxylation, resulting in an elevated NH_3_-N content (Queiroz et al., 2018; Wang et al., 2019). Additionally, microorganisms synthesize microbial protein during fermentation, offsetting the decline in CP content (He et al., 2020). However, the specific mechanism requires further investigation.

### 4.2 Inoculation streamlines the microbial diversity in JA silage, promoting the proliferation of beneficial bacteria

The quality of ensilage fermentation is intricately linked to microbial community changes, with diverse microbial interactions occurring throughout the process (Ni et al., 2017; Wang et al., 2022). This study utilizes high-throughput sequencing for the first time to unveil the distribution of bacteria and fungi in low-temperature ensiled JA. Inoculating with *L. plantarum* results in the lowest bacterial diversity index, signifying a higher proportion of beneficial microorganisms (Du et al., 2021). The fungal Chao1 index shows no significant difference, indicating that inoculation does not affect the richness of the fungal community. At the fermentation’s end, *L. brevis* inoculation yields a lower Shannon index, diminishing fungal community diversity. Factors like low temperature and anaerobiosis inhibit fungal activity, resulting in a gradual succession of fungal communities (Keshri et al., 2018; Cheng et al., 2022). The competition between LAB and undesirable microorganisms decides the success or failure of ensiling, and alterations in the microbial community elucidate the quality disparities among silages (Ni et al., 2017; Bai et al., 2021). Bacterial PCoA illustrates alterations in the bacterial community throughout ensiling, leading to variations in fermentation products (Ni et al., 2017). Inoculating with *L. brevis* results in sluggish bacterial activity, hindering dominant species formation, possibly determined by the inoculant. Conversely, *L. plantarum* inoculation dominates the early ensiling stages, promptly establishing an acidic environment and maintaining stability (Tahir et al., 2023). Fungal PCoA reveals a complete separation of the fungal community on 7 days of ensiling, but no apparent separation on 60 days. After fermentation, raw materials and silage samples intriguingly cluster together. This phenomenon may be because, in the early ensiling stages, aerobic bacteria heavily rely on residual air for reproduction. However, as fermentation advances, low-temperature anaerobic and acidic conditions suppress the activity of aerobic and neutral bacteria, inducing dormancy and eventual stabilization at the same level. The community’s species composition remains unaltered (Bao, 2019). The impact of inoculation on the fungal community composition may not be significant (Duniere et al., 2017; Wang et al., 2022), but specific reasons require further investigation.

Proteobacteria and Firmicutes prevail as bacterial phyla in silages, aligning with our study’s results (Chen et al., 2020; Fu et al., 2023). *L. plantarum* inoculation leads to *Lactobacillus* dominance throughout fermentation, improving low-temperature silage fermentation (Muck et al., 2018; Yan et al., 2019). In contrast to prior studies, *Carnobacterium* and *Desemzia* are notably abundant in both non-inoculated and *L. brevis*-inoculated low-temperature silage of JA. *Carnobacterium*, a Gram-positive bacterium, thrives in frozen soil or decaying plants, displaying facultative anaerobic, cold-tolerant, heterofermentative traits (Leisner, 1992; Meisel et al., 1994; Leisner et al., 2007). *Desemzia*, a thermophilic bacterium, degrades fats, proteins, and simple carbohydrates under aerobic conditions, commonly found in compost fermentation (Zainudin et al., 2017; Qiao et al., 2019). Both bacteria, saprophytes, likely originate from the soil at the base of JA stems and decaying leaves, resulting in silage fermentation failure. The primary fungal phyla in JA silage are Ascomycota and Basidiomycota. Inoculation with *L. brevis* enhances Ascomycota abundance, whereas *L. plantarum* inoculation elevates Basidiomycota abundance. This aligns with Cheng et al.’s (2022) research, highlighting varied LAB effects on fungal communities across distinct feed materials. Regarding genus-level composition, prevalent fungal genera in JA raw materials consist of *Symmetrospora*, *Mycosphaerella*, *Alternaria*, and *Vishniacozyma*. This contrasts with prior research (Gao et al., 2019; Chen et al., 2020; Li et al., 2021), with the disparity linked to distinct raw material types. In the initial fermentation phases, non-inoculated silage samples exhibit dominance of *Vishniacozyma*, *Mycosphaerella*, and *Acremonium* as genera. *Vishniacozyma*, an antagonistic yeast, possesses inhibitory effects against fungal diseases like gray mold (Nian et al., 2023), whereas *Mycosphaerella* and *Acremonium* can induce fungal diseases in plants (Bowness et al., 2016; Hou et al., 2019). This suggests that pathogenic fungi exploit residual air for extensive reproduction in early fermentation stages, prompting allergic reactions and a substantial rise in corresponding antagonistic fungi. *L. brevis* inoculation leads to an increased abundance of *Acremonium*, inhibiting the proliferation and metabolism of *Vishniacozyma*. On the other hand, *L. plantarum* inoculation efficiently inhibits the proliferation of pathogenic fungi but does not suppress the proliferation of *Vishniacozyma* (Santos et al., 2016). The fungal genera mentioned above are infrequently documented in silage. This study reveals interspecies interactions among them in JA silage for the first time. This phenomenon may be linked to the distinctive geographical and climatic features of the Qinghai-Tibet Plateau, along with the rich fructooligosaccharide content in JA stems and leaves. While offering a reference for future investigations into aerobic exposure in JA silage, the specific mechanisms require additional exploration.

LEfSe analysis suggests a positive correlation between biomarker function and the role of community abundance (Barberán et al., 2012; Weiss, 2017). Correlation heatmaps can assess how microbial species affect fermentation parameters, visually illustrating their interaction with species-level biomarkers. *L. plantarum* is positively correlated with LA, while pH and PA show negative correlations, consistent with previous research results (Fu et al., 2023). *L. brevis* is positively correlated with NH_3_-N and AA, while NH_3_-N is negatively or not correlated with Lactobacillus (Wang et al., 2022; Tahir et al., 2023). *L. brevis* is less likely to dominate in silage; its increased abundance accompanies changes in various heterofermentative bacteria, leading to AA synthesis and concurrent NH_3_-N production. The correlation analysis is considered positive, despite changes in heterofermentative bacteria types and continuous NH_3_-N synthesis. Inoculating *L. brevis* did not yield the anticipated results; instead, a significant proliferation of spoilage microorganisms occurred, causing nutrient loss, a considerable increase in NH_3_-N, and possible toxin production, detrimentally affecting livestock consumption. Spoilage microorganisms, including *Carnobacterium inhibens*, *Desemzia incerta*, and *Aerococcus urinaeequi*, show a negative correlation with LA and a positive correlation with pH and PA. As mentioned before, these spoilage bacteria are predominantly found in both non-inoculated and *L. brevis*-inoculated samples, providing additional evidence that *L. brevis* inoculation does not enhance low-temperature fermentation.

## 5 Conclusion

Inoculating low-temperature resistant LAB alleviates the negative impact of low temperatures in JA silage. Inoculating *Lactobacillus plantarum* GN02, which engages in homolactic fermentation, enhances LA levels, lowers pH and PA, sustains an acidic fermentation environment, preserves higher levels of DM and WSC, prevents nutrient degradation, and enhances the quality of low-temperature silage feed in JA. Additionally, it enhances the abundance of *Lactobacillus*, thereby inhibiting the proliferation of detrimental microorganisms like *Carnobacterium*, *Desemzia*, and *Enterobacter*. Inoculating *Lactobacillus brevis* XN25, which undergoes heterolactic fermentation, raises AA levels, but results in a gradual pH decrease. Moreover, it leads to a higher abundance of spoilage microorganisms in silage, consequently compromising fermentation efficacy. In conclusion, *Lactobacillus plantarum* GN02, utilizing homolactic fermentation, exhibits enhanced capabilities for low-temperature fermentation. Therefore, it is the recommended inoculant for winter silage of JA on the Qinghai-Tibet Plateau. This study addresses the deficiency in winter low-temperature silage techniques for JA on the Qinghai-Tibet Plateau. It not only establishes a theoretical foundation for winter silage but also contributes to advancing the development of a green, organic agricultural, and livestock product output center on the Qinghai-Tibet Plateau.

## Data availability statement

The datasets presented in this study can be found in online repositories. The names of the repository/repositories and accession number(s) can be found at: https://www.ncbi.nlm.nih.gov/, PRJNA1013828.

## Author contributions

XW: Writing – original draft. XS: Writing – review & editing. HZ: Writing – review & editing. QZ: Writing – review & editing. GL: Writing – review & editing.

## Footnotes

^1^ chinafeeddata.org.cn

^2^ cloud.oebiotech.com
